# Medical Society of the State of New York

**Published:** 1868-02

**Authors:** 


### Medical Society of the State of New York.

### Sixty-First Anniversary.

FIRST DAY—MORNING SESSION.

The Society met, pursuant to statute, in the State Geological Rooms, Albany, at 11 A. M. on Tuesday, February 4, 1868.

The President, Dr. John P. Gray, of Utica, called the meeting to order, when there being no clergyman present, the Society, on motion of Dr. March, proceeded to regular business.

*Address of the President.*—Dr. Gray next read his inaugural address, and in compliance with the by-law on the subject, gave a sketch of the progress of medicine during the past year. He alluded to the fact that two new medical journals had been started in the city during the time referred to, the *Medical Gazette* and the *Quarterly Journal of Psychology*, and in passing urged the members to sustain all the various medical periodicals by earnest support, both literary and pecuniary. In alluding to the subject of medical education, he deplored the low grade of requirements, and the non-existence of any law which would compel the student to study any specific time. He suggested the propriety of recommending to the legislature, not only the passage of an act naming some particular time for study, but also that the period should be four full years. The question of regulating the proper sale of drugs also came up for consideration, and some legislation was believed to be necessary to accomplish the desired object. Another point made by the speaker in his elaborate, well-timed, and able address, was the necessity of clearing away the stumbling-block to prosecutions for abortions, in the proper understanding of the term "quickening," it being desirable to make all fully amenable to the law who should destroy a fœtus that was viable.

Dr. Bissell, of Utica, offered the following, which was adopted:

*Resolved*, That the President's inaugural address be referred to a committee of three, to examine and report such action thereon as may be deemed necessary on the part of the Society.

The following committees were next appointed:

*Business Committee.*—Dr. E. R. Squibb, of Brooklyn; Dr. S. O. Vanderpoel, of Albany; and J. Foster Jenkins, of Yonkers.

*Committee on Credentials.*—Drs. Hyde, P. P. Staats, and W. H. Bailey.

*Committee on Reception.*—Drs. Allen March, Doolittle, and W. B. Bibbins.

*Committee on President's Address.*—Drs. Brinsmade, Banks, and Rochester.

The Rev. John F. Peck, at this stage of the proceedings, apologized to the Society for his absence at the time of the opening of the session, having been detained some distance from the place of meeting by the sickness of some of his parishioners.

Dr. James H. Armsby offered the following, which was carried

*Resolved*, That a committee of three be appointed to invite the physicians who are members of the Constitutional Convention and the State Legislature to participate in our deliberations during our meeting.

After which Drs. Armsby, Hall, and O. White, were appointed as said committee.

Dr. Bissell announced the death of Drs. John M'Call and Nichol H. Dering, making remarks and offering resolutions.

Prof. White, of Buffalo, made some appropriate remarks in regard to the deceased members.

*Appeal to the Society by an Expelled Member from a County Society.* Dr. Squibb, as Chairman of the Business Committee, presented an appeal from Dr. N. K. Freeman, of West Farms, Westchester county, from a decision of expulsion from the County Society, at the same time suggesting that it would be desirable and proper to refer the matter to a Committee of Censors, who should report their decision to the Society for ultimate action.

The propriety of this measure was discussed, and culminated in the passage of the following resolution, offered by Dr. Bissell:

*Resolved*, That a standing committee on medical ethics, to consist of three members, be annually appointed, to whom all papers, resolutions, and other matters presented to the Society for consideration, involving questions of medical ethics, shall be referred.

Drs. Brinsmade, Hyde, and Banks, were appointed on the aforesaid committee.

Dr. Hun, Chairman of the Committee on the Revision of the By-Laws, read an elaborate report, which was finally referred back, with instructions to report a complete set of by-laws at the next meeting, and have a sufficient number of the same printed for distribution at the commencement of the next annual session.

A communication was next read, inviting the members to an entertainment to be given by Drs. March and Armsby, at the City Hospital, on Wednesday evening, at 9 o'clock.

### FIRST DAY—AFTERNOON SESSION.

The Society met, pursuant to adjournment, at 3 P. M., and was called to order by the President. At the suggestion of Doctor Squibb of the Business Committee, the minutes of the meeting were to be read every morning.

The President announced the following Nominating Committee:

Dr. Jos. C. Hutchinson, Dr. Geo. J. Fisher, Dr. Thos. Hun, Dr. F. Burdick, Dr. D. P. Bissell, Dr. W. C. Wey, Dr. W. Manlius Smith, and Dr. C. C. Wyckoff.

*Legal Enactments to Regulate Practice of Dentistry.*—On motion, Dr. Wescott, dentist, from Syracuse, was solicited to read a report concerning the efforts being made to obtain legislation upon the practice of dentistry. The report was received, and the following preamble and resolution, offered by Dr. Squibb, was adopted:

*Whereas*, The Dental profession of the State of New York, (now numbering about two thousand practitioners,) are about to petition the Legislature of the State for such legal enactments as will tend to regulate the practice of dental surgery, and to mark some distinction between the meritorious and skillful, and the ignorant pretender; and to give this profession a legal recognition, it is by this, the State Medical Soeiety,

*Resolved*, That this movement on the part of the Dental profession of this State, to procure such general laws for their protection as now pertain to the Medical profession, meets with our hearty approval, and that wehereby join in the prayer of these petitioners for this purpose.

*The Sulphite of Soda and Glycerine.*—Dr. John H. Griscom, of New York, read a paper entitled "Therapeutic Value of Certain Articles of the Materia Medica of Recent Introduction," in which he treated more particularly of the uses of sulphite of soda as an internal, and glycerine as an external remedy. The former, by its peculiar antiseptic property, was considered by him as one of the most valuable alteratives, especially in those cases of functional dyspepsia, where it was necessary not only to arrest the fermentative process, but also to furnish an alkali. The latter substance, in consequence of its affinity for the watery elements of the blood, had, according to his observations, a marked effect in melting down all inflammatory indurations.

The paper was, on motion, referred to the Publication Committee, not however without some humorous allusions by Dr. Corliss to hobbyism in new remedies generally.

Dr. Marsh introduced Prof. Barker, of New Haven, formerly of Albany. Prof. Barker gracefully responded to the introduction. Also Charles L. Ives, of New Haven, who also addressed the Society; as did also Drs. Foster, of Portland, Maine, and Lewis A. Pendleton, of Maine.

Dr. Squibb read by title the following papers: "Method in Medicine," presented by the Albany County Medical Society, by Dr. Pomfret; continuation of paper on "Monstrosity," by G. J. Fisher, of Sing Sing; all of which were referred to the Publication Committee.

FIRST DAY—EVENING SESSION.

The Society met at 8 P. M., and was called to order by the President.

Dr. Squibb presented the "Biographical Sketch of Alden S. Sprague, M. D.," by C. C. Wyckoff; "A Report from the Madison County Medical Society, upon some of the Diseases of that County," by Dr. Saunders. All of which were referred to the Publishing Committee.

Dr. Garrish read his report on "Tsan-Tsi or Rhynchosa Excavata in Amenorrhœa." Referred to Publication Committee.

Dr. Wolff moved to lay the whole subject on the table. Lost.

Dr. Garrish next presented a specimen of an acephalous monster.

Dr. Bates, from Columbia county, made a report as delegate to the Massachusetts Medical Society; after which the Society adjourned until Wednesday, 9½ A. M.

SECOND DAY—MORNING SESSION.

The meeting was called to order by the President, Dr. Gray, when the Rev. Mr. Smart, of Albany, offered a prayer. The minutes of the meetings of the previous day were read and approved.

Dr. Coventry, of Utica, presented a memoir of the late Dr. John M'Call, which was referred to the committee on publication.

*The Uses of the Sphygmograph.*—Dr. S. O. Vanderpoel, of Albany, exhibited, by request, the workings of the sphygmograph.—He stated that Dr. Hun, of Albany, was the first gentleman who had brought the instrument to this country, and that with him he had tested its extended applicability in a large number of cases. After stating that the instrument was destined to do for cardiac disease what the stethoscope had done for chest troubles, he described the manner in which the pulse-waves were registered, and the significance of the tracings.

Dr. March introduced Dr. Hitchcock, a delegate from the Massachusetts State Society.

Dr. Allaben, of Delaware county, read a report of a case of compound fracture of the leg, which had occurred in that county, and for the treatment of which he had been sued in the sum of $500 for mal-practice, in causing mortification, as was claimed, by tight bandaging. The facts of the case, as they appeared in the report, were adverse to such a decision. His object in presenting the case to the Society was to obtain some expression of opinion, not only upon this case, but upon similar ones. The report was referred to the Committee on Publication.

On motion of Dr. Lee, of Peekskill, the whole matter was referred to the following committee of three: Drs. Armsby, Hutchinson, and Wolcott.

*Anastomosing Aneurism of the Orbit.*—Dr. Armsby introduced the Rev. Dr. Hartwell, who, a year before, had been seized with an inflammation of the left eye, terminating in a slight protrusion of the eye-ball, which had gradually been increasing until the present. The tumor was strongly pulsating in character, and could be diminished somewhat by pressure upon the carotid of that side. The diagnosis had been that of ophthalmic aneurism. The question as to an operation of ligation of the carotid came up, and would in all probability have been performed, had not an aneurismal varix suddenly made its appearance upon the prominence of the left shoulder. The patient's health was beginning to break down, and the expression of opinion on the part of the members was solicited as to the propriety of any operative interference.

Dr. March gave it as his opinion that the disease was an aneurism by anastomosis, and believed with others present that the case was a hopeless one for surgical benefit.

Dr. Quackenbush read the Treasurer's Report, showing a balance of over one hundred dollars to the credit of the Society.

*Holt's Instrument in Stricture* —Dr. Hutchison, of Brooklyn, read a paper on the uses of Holt's instrument in the treatment of stricture, and cited some illustrative cases. As this paper will be presented to our readers at some future time, we shall not here make an abstract of it.

Dr. March related a case of cancer of the rectum, for which he had removed two inches of the gut. This case will also probably be published *in extenso* at a future time.

*Report of Committee on President's Inaugural.*—Dr. Brinsmade, as Chairman of the Committee on the President's Inaugural, offered the following, which were unanimously adopted:

The committee to whom so much of the President's address was referred as pertains to legislative action, respectfully report:

*First*—That in their judgment, laws should be enacted, if possible, to secure proper qualifications and restrictions, on the part of those to whom the preparation, compounding, and sale of drugs and medicines is entrusted.

*Second*—That criminal abortion is of such frequent occurrence, and is so lightly regarded by the community in general, that its wickedness and enormity, and destructiveness to health, can only be made apparent by the united expression of the medical profession; and that it is the sense of the Medical Society of the State of New York, that the words "with a quick child," in chapter 22, § 1, quoted in the address, should be stricken out, and the statute thus amended made to cover the whole period of gestation.

*Third*—That the remarks on the preliminary education of students, and on their proper medico-collegiate course, are most fully and thoroughly endorsed, and that all legitimate action to secure the same is most earnestly commended.

*Fourth*—Without further specification, the committee advise the publication and wide dissemination of the address, as presenting the sentiments of the Society and of the profession at large; and lastly, they recommend the appointment of a local committee to present, if practicable, the necessary documents to the present Legislature of the State of New York, to procure such legal enactments as shall accomplish the end desired—and if that cannot be done, to present the same to the next Legislature at the commencement of its session.

THOS. C. BRINSMADE,

JAMES F. BANKS,

THOS. F. ROCHESTER.

Dr. Corliss offered his report, as delegate to the Rhode Island and Vermont Medical Societies.

The following committee on the above resolutions of Dr. Brinsmade, as Chairman of the Committee on President's Inaugural, was announced: Drs. Brinsmade, Banks, and Rochester.

*Microscopical Examination of Damaged and Valuable Papers.*—Dr. E. H. Parker, of Poughkeepsie, remarked on the use of the microscope in detecting alterations in valuable papers. The remarks were based upon two very interesting cases of forgery, to which the doctor had been called as an expert.

The first was one in which it was an alleged promissory note, signed by a blind man who had deceased. The gentleman in question had become blind by cataract, but was nevertheless in the habit of signing all important papers. The body of this note was written by a different hand, in blue ink, and the name in black ink. The question came up as to whether the body of the said note was written before that of the signature or not.

The paper folded end to end across the middle. Prints of black ink were transferred from the black signature, and were found on the opposite side. In several places the blue and black ink of the dots were in conjunction. It was impossible to tell which was put on last, till a place was found where the bottom of a letter *y* and tip of a letter *h* came together over a dot, and showed the blue ink on top.

The same paper read, "*one day* after my death I promise to pay," etc. It showed clearly under the microscope that it had been written "one *year*," an erasure having been made and *day* written in.

The other paper was an alleged receipt for $2,000, paid on May 11th. That amount had been paid May 1st, and this alleged payment was denied to have been made. Examination by microscope showed that the first figure 1 of the date was in *brown*-black ink, while the second and the rest of the paper were in *blue*-black ink. Transfer had been made of the *brown*-black ink to the other end of the paper by folding, showing that it was put on last. The two shades of black show only under the microscope; to the naked eye they are alike.

Dr. Squibb, in this connection, referred to the following case: A number of U. S. bonds were stolen some time since from a party, and their payment stopped. For a long period nothing could be discovered in relation to them. Finally, however, two bonds with the same numbers were fouud in Wall street, and it occurred to the parties concerned that one of these must be of the lot that had been stolen. The difficulty was to decide which was the genuine, and it was cleared up by a microscopical examination of the ruled lines upon which the figures were written in red ink. The magnifying glass showed the tracings of the old figures underneath the new, the red ink of the former having been previously removed by a chemical process.

Dr. R. H. Ward, of Troy, next read a very lengthy paper with the following title: "Allopathy; an Inquiry into the Relation of Sects in Medicine."

Dr. Corliss related a case of ovarian dropsy which he had tapped through the vagina.

Dr. Squibb, in behalf of the Business Committee, stated that information had been received from Dr. Ives, of New Haven, that Elial T. Foote, a permanent member of the Society, had been a declared charlatan for many years. The said party was a resident of New Haven, and had done much, with such influence as he possessed, to damage the interests of the profession at that place.

A motion was accordingly made to drop the said name from the roll, which, after much discussion, was carried.

The Society then adjourned until 3½ P. M.

SECOND DAY—AFTERNOON SESSION.

The Society met at 3½ P. M., agreeably to adjournment. The meeting was called to order by the President.

Dr. Cobb moved that the Society request the Nominating Committee to present the name of Dr. Corliss for our next President. This was lost.

Dr. W. White made his report as delegate to the Maine Medical Society, which was referred.

Dr. Squibb presented the report of Dr. A. N. Bell, from Committee on Hygiene. Referred.

*A Worm in the Aorta. (?)*—Dr. Coates, of Batavia, presented a portion of the arch of the aorta containing a lumbricoid which was discovered in that artery during a post-mortem examination of a soldier in the Ladies' Home Hospital, of New York city. The patient had died in 1864 of pneumonia, following an attack of measles. On attempting to remove the lungs by severing the vessels at their roots, the worm referred to was found alive in the cavity of the aorta. It was preserved *in situ*. The gentleman' Dr. Tozier, who forwarded the specimen, not having sent a complete history of the case, Dr. C. was unable to give any further particulars.

Dr. Squibb pertinently remarked at this juncture, that in all probability the worm had accidentally found its way into the interior of the vessel after the death of the patient.

The Business Committee read by title a paper entitled, "Removal of Encephaloid Testicle," by Dr. Ferris Jacobs, and one on "Cæcal Inflammation and Ulceration complicated with Diseased Appendix," which were referred.

*Case of Inverted Action of Uterus.*—Dr. P. P. Staats, of Albany, reported a case of foot presentation, which was accompanied with repeated and forcible recession of the parts. The feet were finally delivered, when a large retained placenta was found at the fundus.

Dr. Squibb suggested that the placenta in that situation, acting as a splint, so to speak, interfered with the action of the uterine fibres, and gave a chance for those below to press the lower parts of the child upward.

Dr. Wm. B. Bibbins offered the following resolution:

*Resolved*, That a physician of this State will not be received by this Society as an invited guest, unless he is either a member of a county medical society, or a member of the New York Academy of Medicine, or a member of the faculty of a medical college, or a member of the legislature.

Adopted.

Dr. White, of Buffalo, offered the following, which was adopted:

*Whereas*, Observations have been made, during an informal discussion yesterday in this Society, questioning the propriety of continuing the publication of the essay of Dr. G. J. Fisher on "Compound Monsters;" and *whereas*, Dr. Fisher in his remarks intimated that he should "feel delicate about offering to the Society anything further on the subject."

Therefore *Resolved*, That this Society recognizes the scientific value of this essay, and earnestly requests Dr. Fisher to continue his monograph, and furnish it to the Committee of Publication.

Dr. Beadle moved the following: That the Committee on Pharmacology be directed to report to this Society the name of a member to supply the place in that Committee of Dr. Townsend, deceased. Adopted.

Dr. Squibb moved that the Society meet Thursday morning, at 9½ A. M.

On motion, the Society adjourned, to meet in the Assembly Chamber at 8 o'clock, to hear the President's Address.

The Society convened in the Assembly Chamber at 8. They were called to order by Dr. Tefft, Vice President; after which the President, Dr. Gray, delivered his annual address.

*The Annual Address.*—The subject which had been chosen was the relation of the study of insanity to general medicine. After showing that insanity was properly a branch of general medical science, he gave an historical review of the progress which had been made in this study; and proved that it had been in proportion to its study as a branch of medicine rather than of mental philosophy. He stated that the ancients regarded it as a bodily disease, hence they were tolerably correct in their treatment; but during the middle ages it fell within the domain of philosophy, and was consequently misunderstood. In modern times its progress in Europe and this country had been due to the investigations of medical men taking it from the control of metaphysical speculators. He remarked that the history of the subject showed that, freed from the above-noted retarding influences, its progress had kept pace with that of general medicine, of which it was legitimately a part. Insanity was now recognized as a physical disease, and it should be taught as such in medical schools. The advantages of such teaching were then dwelt upon, after which some practical hints were offered as to how such a plan might be carried out.

It was moved by Dr. Van Dyke that the thanks of this Society be tendered to Dr. Gray for his very able and interesting address. Adopted.

The Society adjourned, to meet at 9.30 A. M. Thursday.

After the adjournment, the members assembled at the City Hospital, to respond to the invitation to an entertainment by Drs. March and Armsby. Telling addresses were made by Mr. Bogart, the Hon. Mr. Brooks, and others, after which all present partook of a bounteous and elegant supper.

(To be continued.)

